# Social media provides support and education for pregnant people when healthcare does not

**DOI:** 10.3389/fgwh.2024.1410831

**Published:** 2024-11-28

**Authors:** Elissa Z. Faro, Donna A. Santillan, Meghan L. Funk, Kara Boeldt, Mark K. Santillan

**Affiliations:** ^1^ Internal Medicine, University of Iowa Hospitals & Clinics, Iowa City, IA, United States; ^2^ Obstetrics & Gynecology, University of Iowa Hospitals & Clinics, Iowa City, IA, United States; ^3^ EndPreeclampsia, LLC, Chicago, IL, United States

**Keywords:** Preeclampsia, social media, advocacy, mental health, pregnancy

## Abstract

**Introduction:**

The use of social media for health-related reasons is growing, but there is a dearth of research on the mechanisms of support provided. Understanding how social media groups work could improve communications between providers and patients. Preeclampsia (PreE) is a hypertensive disease of pregnancy that has short- and long-term physical and psychosocial effects. The Preeclampsia, Eclampsia & HELLP Syndrome Survivors Global Support Network (PEHSS) Facebook group is an online, international, moderated support group that provides evidence-based information and community support. Our study aimed to (1) characterize the forms of social support and types of information sought and provided from the perspective of the group moderators and members, and (2) describe group members' experiences of patient care. We triangulated interview and survey findings to identify gaps in care, ultimately to inform in improvements in care delivery.

**Methods:**

We began with 30–45-minute semi-structured interviews with PEHSS moderators exploring experiences and perceptions of membership; preliminary findings were member-checked with additional moderators. Interviews were analyzed using template and matrix analysis. Based on emergent themes, we conducted an online, validated patient experience survey with PEHSS members that was analyzed using descriptive statistics.

**Results:**

Emotional and social support, mental health, resources and education, and personal health advocacy emerged as major themes in the 12 interviews. 1,148 PEHSS members responded to the survey. 68% of survey participants wanted to be more involved in the decisions about their care and treatment and over 30% felt they were not informed about danger signals post discharge while approximately half reported always feeling treated with respect and dignity while in the hospital. Geographic analysis showed differences in experiences of communication with providers within and outside the US.

**Discussion:**

The triangulated results from interviews and surveys indicated a need for better communication with providers and the ability for patients to have more input on their care. The survey results indicate a global issue in providing support for people with hypertensive disorders of pregnancy during their hospitalization. The needs currently supported through communities on social media highlight opportunities to address critical gaps in care.

## Introduction

Preeclampsia (PreE), a hypertensive syndrome in pregnancy, affects 5%–7% of all U.S. pregnancies and is a leading cause of worldwide obstetric mortality with nearly 600,000 associated annual deaths (1). Improved diagnosis and prediction of PreE have been shown to improve future prevention of short-term and long-term cardiovascular disease in women (2, 3). Research has shown that hypertensive disorders of pregnancy have long-term impacts on mental health, including higher rates of psychiatric disorders including depression, post-traumatic stress disorder (PTSD), and anxiety (4). PreE is a prime example of a disease in pregnancy the health effects of which span the lifetime; it is known to be associated with immediate and long-term maternal-fetal morbidity and mortality (5–10).

Social support is a well-documented and well-studied aspect of pregnancy and the transition to motherhood, providing a critical source of information, emotional empathy, and understanding (11–14). The ubiquity of the online environment, along with changing family structures and increasing numbers of single parents, working parents, and parents who do not live near extended family, contribute to the creation of new models of social support which incorporate social media networks (15). Research has shown that pregnant people use the internet to seek social support from other pregnant people; to research specific problems; for advice on home remedies; to take part in discussion groups; and for information on antenatal tests for birth anomalies (16). In particular, one study demonstrated that midwife-moderated Facebook groups provided safe spaces for sharing and social support as well as sources of maternally-relevant, reliable information and significantly impacted perceptions of relational continuity (17).

Social media is an increasingly relevant form of support and source of health-related information that can impact pregnant people's health-related behavior and mediate their relationship with the healthcare system. One systematic review of social media use for health-related reasons found it can affect the relationship between patients and healthcare professionals; social media leading to patient empowerment can result in more equal communication between the patient and healthcare professional and improved decision making (18). Perinatal healthcare professionals have an opportunity to learn from the types of support and information sought through social media to improve communication and education (19, 20).

A private Facebook group—Preeclampsia, Eclampsia & HELLP Syndrome Survivors Global Support Network (PEHSS)—supports pregnant people and their community specifically around hypertensive disorders of pregnancy. PEHSS membership has grown rapidly; in 2019 it had 28,000 members and now has over 46,000. PEHSS is a global, evidence-based support, awareness, education, and advocacy group for those who have been affected by Preeclampsia, Eclampsia, HELLP Syndrome, Gestational Hypertension, Chronic Hypertension or any hypertensive disorder of pregnancy. PEHSS welcomes and supports new or suspected cases/diagnosis, those with a diagnosis, those who have previously had a hypertensive pregnancy, loved ones of an affected person, health care providers, researchers, and journalists. The advice and information provided by the moderators of the group is based on American College of Obstetrics and Gynecology Taskforce on Hypertension Guidelines (21), the National Institute for Health and Care Excellence guidelines (22), and other similar guidelines around the world. PEHSS is education- and learning-based and focuses on providing data-driven information as well as emotional support to members. The group is administrated and moderated by trained PreE Educators & Patient Advocates, all of whom have personal experience with hypertensive disorders of pregnancy. The group moderators vet all questions and comments from group members, who are very active: on average, PEHSS has 80% engagement of members, 1,500 posts, and 15,000 member comments per month. PEHSS also engages members through Facebook live events with medical and educational content experts to answer members' questions around a particular theme (e.g., long-term health effects of PreE).

The objective of our research is to understand the mechanisms of the PEHSS group by qualitatively characterizing the forms of social support and types of information sought and provided from the perspective of the group moderators and members and quantitively describing group members' experiences of patient care.

## Materials and methods

### Study design

Our mixed methods ethnographic study consisted of semi-structured interviews, the findings of which were member checked, and a patient experience survey to capture the breadth and depth of experiences of people with PEHSS globally. The study was determined exempt by the University of Iowa Institutional Review Board (protocol #202012328).

### Study setting and population

At the time the research was conducted, the PEHSS group had a global membership of over 37,000 people in 115 countries. On average, 83 new members join per day and there are over 50 new posts and 500 comments daily, requiring 1,000+ volunteer hours per month to manage.

### Data collection

#### Semi-structured interviews

The research team (EF, DS, KB, MS) developed a six-question interview guide consisting of questions about moderators' experiences with the PEHSS group, their opinions about the experiences of the members of the group, and questions to elicit their ideas about how to improve communications with group members. The focus was on the benefits and challenges for members of the PEHSS group and the means by which the group provided unique support mechanisms for participants. Interview question included: “What do you think are the benefits for members of the preeclampsia group?”; “Do you think there are any challenges with being a member of the group?”; “Can you think of anything that would help you better moderate the group?”; and “What do you think would be most helpful for participants?”. Typically, the interviews lasted 30–45 min and were conducted virtually via Zoom. We also shared the questions via email with moderators upon request if we weren't able to schedule an interview. Interviews were video- and audio-recorded and transcribed for analysis.

#### Member checking

Two discussions were held virtually in August 2021 with the moderators to facilitate participant validation by presenting the preliminary results of the semi-structured interviews for feedback (i.e., member checking) (23), and to brainstorm potential interventions to address some of the issues identified. The discussions were held via Zoom, video- and audio-recorded, and transcribed for analysis.

#### Patient experience survey

After the interviews and discussions, the research team sought to confirm and explore more broadly the initial findings with the entire membership of PEHSS. We invited all 37,000 members of the PEHSS group to complete a validated survey instrument online through posts on the group's social media accounts: Facebook (FB), Instagram, and Twitter. The instrument, the Picker Patient Experience questionnaire, asks about participants' experience with birth in hospital around eight dimensions: information and education; coordination of care; physical comfort; emotional support; respect for patient preferences; involvement of family and friends; continuity and transition; and overall experience of care (24).

To facilitate recruitment, we (1) hosted a FB Live and a Live Room (i.e., built-in Facebook capability to livestream broadcasts to an audience) to announce the survey and answer any initial questions (25); (2) posted and pinned the FB Live recording to the announcements section of the group's page; (3) posted a link to the survey and the FB Live recording in the group's Research Learning Guide (i.e., the section of PEHSS' facebook page where resources are available) 1–5 times daily (5 a.m., 11 a.m., 3 p.m., 8 p.m., 11 p.m.) utilizing a social media management platform, Loomly; and (4) posted the FB Live recording at least once a day at various times utilizing Loomly (26, 27). Potential participants clicked a link in the social media post that sent them directly to a University of Iowa Hospitals and Clinics-hosted REDCap survey with consent language. The survey was open for 1 month in November 2021.

Participants answered a brief demographic questionnaire (specific to pregnancy, birth, and location) to determine eligibility and completed the Questionnaire (24). Altogether, the survey took approximately 10 min to complete, and participants were able to skip any question at any time. 1,169 members answered at least one question in the survey.

### Data analysis

Using an ethnographic approach, the interview data were analyzed using template and matrix analysis (28–30) to develop a descriptive and contextualized understanding of the experience of both moderators and members of the PEHSS group regarding communications, needs and preferences around hypertensive disorders of pregnancy, and healthcare generally. The research team developed and piloted a template based on the domains in the semi-structured interview guide. Once the template was finalized, each interview transcript was independently summarized by two team members. Each transcript summary was compared and discussed, and discrepancies were resolved by consensus (31). Data from the summarized transcripts were used to build a matrix arranged by *a priori* domains (31). The research team met to analyze the matrix and identified key themes that were used to facilitate the member-checking discussions. The discussion transcripts, together with notes taken during the discussion, were analyzed using thematic analysis. The findings were discussed by the research team to inform the selection of the survey instrument.

The survey data were analyzed using descriptive statistics to understand general patterns in experience across the PEHSS group membership. We also analyzed geographic variation using Chi square tests for categorical variables (i.e., delivery method, Picker scores) and compared results from United States (US) participants and those outside of the US. Student's *t*-tests were utilized for continuous variables such as maternal age and years since pregnancy. In cases where criteria for normality were not met, Mann-Whitney tests were utilized. All variables were tested at a significance level of 0.05. SigmaPlot 14.5 was used for analyses.

## Results

### Interview results

Between March and May 2021, we conducted nine interviews with the trained Preeclampsia Educators & Patient Advocates who are active moderators of the PEHSS group and received responses from three additional participants via email. The findings from the research team's matrix and thematic analysis were honed further by the discussions with PEHSS moderators. We present below the qualitative findings in four overarching themes: (1) emotional and social support; (2) mental health; (3) resources and education; and (4) personal health advocacy. While emotional and social support, mental health, and personal health advocacy focus on what the PEHSS group provides members, and resources and education focuses more on member needs, all four themes identify critical gaps in care, information, and support experienced by PEHSS members.

#### Emotional and social support

The most important thing moderators felt was provided by the PEHSS group was emotional and social support—the connection, community, and a sense of not feeling alone. Feelings of isolation and lack of understanding from family and friends, despite their concern, was one of the biggest challenges of their experience with PreE cited by interviewees.

“…because being a loss mom [a parent who has lost a child] is the hardest thing. And it changes you. Sorry, it changes you as a person… It changes as a person, it changes you as a mother, changes you as a wife, as a friend… And what I learned was that actually other mums are really important to have lived the experience that you've lived because no one else understands. They don't. And they can tell you that they do. And they can show you so much love and so much support. But the shoes that we walk as loss moms. Just painful. And unless you've walked them, people don't understand.”

The moderators identified the biggest benefit for members of the group, and themselves, was being a community that addressed this isolation in addition to providing education, resources, and other advocacy materials. One moderator described this sense of community that made the group unique,

“As much as this group is for education and advocacy for preeclampsia, eclampsia, and HELLP Syndrome, I think the positive social supports from members and moderators and the connectivity this group enables between members and experiences is a unique quality that makes it stand out.”

Another moderator explained in more detail,

“I think the largest benefit is social connection with others with shared experiences, self-paced education, documents to help advocate at doctors visits (regarding plan of care, questions to ask), and being within a community with 24-h moderator support.”

In general, the moderators felt that having a carefully curated space, such as ensuring that a trained person be the first to respond to a post or that resources shared were all evidenced-based, was integral to creating a supportive community space.

#### Mental health

Confirming research that people with hypertensive disorders of pregnancy have higher rates of psychiatric disorders including depression, post-traumatic stress disorder (PTSD), and anxiety (4), moderators identified mental health as a major challenge for members of the PEHSS group.

“I will say that I wish my doctors were more proactive about mental health discussions and offered me support during my hospital stay or early postpartum. I do wish there were more conversations with my medical team about trauma and PTSD. I truly feel that trauma and PTSD get overlooked by PPD/PPA [postpartum depression/postpartum anxiety]. It was too hard for me to initiate conversations about my experience which is why I waited as long as I did to seek help.”

Given the prevalence of mental health issues among the group, while sharing experiences was seen as one of the main benefits for members of the group, moderators described how this could be a trigger for many members with PTSD, anxiety, and trauma.

“We will have some weeks where we will have five or six deaths, either baby deaths, maternal deaths on the boards and you're like it's just overwhelming. And depending on the stage that you are in, if you are pregnant and anxious, and that's just already skyrocketing your anxiety level because you're sitting there going, this person's story isn't much different from mine. Am I going to die? Am I gonna lose my baby? And I think that is a really hard challenge because we spend a lot of time telling moms it's normal that you're anxious. You are having anxiety symptoms. You need to talk to your doctor about looking and treating this anxiety.”

In addition to identifying the need for perinatal mental health support generally, noting this lack the moderators also felt that having a trained mental health professional available on the PEHSS FB platform would be beneficial for members as well.

#### Resources and education

Moderators talked about the importance of resources and education for PEHSS members, in particular enhanced access to resources and information for the members but also for the moderators themselves.

“I've seen over and over again, medical professionals not explaining properly what's going on, not explaining the care, not explaining what's going on to these women about what's happening in their own bodies, what they're expecting, or what to expect. And time after time, we are filling that gap.”

Given all the information available on the internet, it was also suggested that training on how to understand research and assess the evidence-base of information would be helpful.

“*Reader-friendly materials* containing the knowledge and research within the learning guides (in addition to all the research papers and abstracts in the learning guides already there). I feel that the bulk of reading can be difficult for some, and seeing that this is a global group, I think it's important to make sure that we can share the evidence- based practices and key information in ways that can be easily understood and legible by members of various backgrounds and statuses (thinking cognitively, receptively).”

The moderators recognized and supported the policies of the PEHSS Group to share evidence-based practices and the most current literature, but also recognized that they were there to support members in their own health and healthcare journeys with the medical world as well.

#### Personal health advocacy

Moderators explained how much of their work focused on teaching personal health advocacy to support members in talking to their healthcare team.

“Which is why a lot of what we do is helping to teach people to advocate for themselves. And even just teaching them the proper terminology to use to help better that communication between themselves and their providers.”

Another moderator explained:

“I think one would help maybe is like more guidance, maybe on talking points with your doctor. You know, sometimes like we offer the information. But there's a bigger question about how do I go about discussing this with my provider, you know, and why do I do when my providers like you're fine, you have nothing to worry about. Like this is not important. And you know that it's important because you read about it. But there's this this whole area. But advocating for yourself, that is hard, I think.”

While the moderators described how they and other members provided support for how to communicate and advocate for themselves, they also expressed a desire for improved healthcare professionals' capacity to communicate and support patients and their families.

### Questionnaire results

1,169 PEHSS members responded to the online survey; not all participants chose to answer every question. The majority of participants identified as female, white, heterosexual, living in an urban area, employed full-time, and have a bachelor's degree (Table 1). Most participants were from the US (*n* = 909, 79.8%), United Kingdom (*n* = 58, 4.6%), Canada (*n* = 51, 4.1%), and Australia (*n* = 36, 2.9%) (Table 2). There were statistically significant differences in birth outcomes, marital, and employment status between US participants and non-US participants. Results from the Picker Patient Experience questionnaire were initially pooled (US + non-US participants) to identify broad patterns across PEHSS membership regardless of intersecting identities. We then subset the data to analyze differences between participants from within and outside of the US given that not only healthcare practices but also guidelines and other policy-related issues vary by country and region (Table 3).

**Table 1:** Survey participants’ demographics by location.

Characteristic	United States	Outside of US	*p*-value
	*N* (%) or Average ± SD or as noted	*N* (%) or Average ± SD or as noted	
	*N* = 909	*N* = 260	
Age at survey completion (years)	33.7 ± 6.7 years	32.16 ± 6.2 years	0.8
Number of years between affected pregnancy and survey completion	3.5 ± 4.9 years	3.2 ± 4.9 years	0.5
Number of pregnancies (median, 1st IQR, 3rd IQR)	2, 1, 3	2, 1, 3	0.3
Number of living children (median, 1st IQR, 3rd IQR)	1, 1, 2	1, 1, 2	0.2
Outcome of the most recent pregnancy affected by preeclampsia, eclampsia, or HELLP			
- Live birth	851 (94.3%)	221 (86.3%)	<0.001
- Pregnancy loss before birth	8 (0.9%)	4 (1.6%)	
- Stillbirth	23 (2.6%)	14 (5.5%)	
- Baby loss after birth	20 (2.2%)	17 (6.6%)	
Area of residence			
- Rural	123 (13.5%)	Unable to determine	N/A
- Urban	761 (83.7%)		
- Unknown (Zip code not provided)	25 (2.8%)		
Gender			
- Female	905 (99.8%)	256 (99.2%)	0.2
- Do not identify as male or female (gender non-conforming, gender queer)	2 (0.2%)	1 (0.4%)	
- Transgender	0 (0%)	1 (0.4%)	
- Prefer not to answer or blank	2 (0.2%)	2 (0.8%)	
Sexual orientation			
- Lesbian, gay, or homosexual	2 (0.2%)	1 (0.4%)	0.5
- Straight or heterosexual	842 (93.5%)	234 (92.1%)	
- Bisexual	48 (5.3%)	13 (5.2%)	
- Something else	1 (0.1%)	1 (0.4%)	
- Prefer not to answer	8 (0.9%)	5 (2.0%)	
Education			
- Less than a high school diploma	6 (0.7%)	4 (1.7%)	0.4
- High school degree or equivalent	174 (20.5%)	51 (21.2%)	
- Bachelor's degree	326 (38.4%)	100 (41.9%)	
- Master's degree	250 (29.5%)	69 (28.6%)	
- Doctorate	83 (9.8%)	16 (6.6%)	
- Other	9 (1.1%)	1 (0.4%) (1)	
Marital status			
- Single (never married)	50 (5.5%)	17 (6.6%)	<0.001
- Married	802 (88.3%)	189 (73.5%)	
- In a domestic partnership	29 (3.2%)	42 (16.3%)	
- Divorced	27 (3.0%)	6 (2.3%)	
- Widowed	0 (0%)	3 (1.2%)	
Current employment status			
- Employed full-time (40+ h a week)	518 (57.7%)	124 (48.8%)	<0.001
- Employed part-time (less than 40 h a week)	134 (14.9%)	62 (24.5%)	
- Unemployed (currently looking for work)	12 (1.3%)	9 (3.5%)	
- Unemployed (not currently looking for work)	159 (17.7%)	26 (10.2%)	
- Student	22 (2.5%)	11 (4.3%)	
- Retired	5 (0.6%)	2 (0.8%)	
- Self-employed	48 (5.4%)	20 (7.9%)	

**Table 2:** Survey respondents’ geographic location.

Country	Participants (*n*)	% of respondents
United States	909	80.3
United Kingdom	58	5.1
Canada	51	4.5
Australia	36	3.2
Germany	12	1.1
Afghanistan	7	0.6
Sweden	6	0.5
New Zealand	5	0.4
Kenya	4	0.4
Netherlands	4	0.4
Finland	3	0.3
France	3	0.3
Philippines	3	0.3
Honduras	2	0.2
Brazil	2	0.2
Cyprus	2	0.2
Nigeria	2	0.2
Norway	2	0.2
Pakistan	2	0.2
Zambia	2	0.2
Other (1 person per country)	15	0.1 each

**Table 3:** Participants' Picker survey responses.

Questions and possible responses	Survey respondents	US participants	Non-US participants	*p* value
	100%	% (*n*)	% (*n*)	
When you had important questions to ask your provider, did you get answers that you could understand?				
- Yes, always	37.8% (433)	40.6% (368)	27.6% (71)	<0.001
- Yes, sometimes	49.1% (562)	47.5% (431)	54.1% (139)	
- No	11.8% (135)	10.7% (97)	16.7% (43)	
- I had no need to ask	1.2% (14)	1.2% (11)	1.6% (4)	
When you had important questions to ask a nurse, did you get answers that you could understand?				
- Yes, always	32.7% (373)	34.5% (313)	25.4% (65)	<0.001
- Yes, sometimes	53.8% (614)	53.7% (487)	53.9% (138)	
- No	12% (137)	10.7% (97)	17.2% (44)	
- I had no need to ask	1.6% (18)	1.1% (10)	3.5% (9)	
Sometimes in a hospital, one provider or nurse will say one thing and another will say something quite different. Did this happen to you?				
- Yes, often	25.5% (291)	23.1% (209)	35.0% (90)	<0.001
- Yes, sometimes	42.1% (481)	43.9% (397)	35.8% (92)	
- No	32.4% (370)	33.1% (299)	29.2% (75)	
If you had any anxieties or fears about your condition or treatment, did a provider discuss them with you?				
- Yes, completely	29.7% (340)	30.3% (275)	26.5% (68)	0.11
- Yes, to some extent	48.3% (552)	49.0% (444)	45.9% (118)	
- No	19.2% (219)	18.3% (166)	23.7% (61)	
- I didn't have any anxieties or fears	2.8% (32)	2.4% (22)	3.9% (10)	
Did providers talk in front of you as if you weren't there?				
- Yes, often	8.7% (99)	7.5% (68)	12.5% (32)	<0.001
- Yes, sometimes	23.2% (265)	21.3% (193)	31.1% (80)	
- No	68/1% (778)	71.2% (645)	56.4% (145)	
Did you want to be more involved in decisions made about your care and treatment?				
- Yes, definitely	34.6% (395)	33.3% (301)	41.6% (107)	0.01
- Yes, to some extent	33.5% (382)	33.1% (300)	34.3% (88)	
- No	31.9% (364)	33.6% (304)	24.1% (62)	
Did you feel you were treated with respect and dignity while you were in the hospital?				
- Yes, always	54.1% (619)	54.4% (493)	52.3% (135)	0.50
- Yes, sometimes	35.0% (400)	35.1% (318)	34.5% (89)	
- No	10.9% (125)	10.6% (96)	13.2% (34)	
Did you find someone on the hospital staff to talk to about your concerns?				
- Yes, definitely	35.6% (407)	36.4% (330)	31.8% (82)	0.40
- Yes, to some extent	34.8% (398)	33.9% (307)	38.4% (99)	
- No	22.0% (251)	21.9% (198)	23.2% (60)	
- I had no concerns	7.6% (87)	7.8% (71)	6.6% (17)	
Were you ever in pain?				
- Yes	80.8% (923)	80.7% (731)	80.9% (208)	0.93
- No	19.2% (219)	19.3% (175)	19.1% (49)	
Do you think the hospital staff did everything they could to help control your pain? (if question above = yes)				
- Yes, definitely	55.7% (512)	57.4% (419)	48.8% (100)	0.03
- Yes, to some extent	28.4% (261)	28.4% (207)	30.2% (62)	
- No	15.9% (146)	14.2% (104)	21.0% (43)	
If your family or someone else close to you wanted to talk to a provider, did they have enough opportunity to do so?				
- Yes, definitely	33.2% (379)	35.1% (318)	24.9% (64)	<0.001
- Yes, to some extent	39.6% (452)	40.2% (364)	38.5% (99)	
- No	16.4% (187)	14.1% (128)	24.9% (64)	
- No family or friends were involved	3.0% (34)	2.4% (22)	5.4% (14)	
- My family didn't want or need information	6.2% (71)	6.5% (59)	4.7% (12)	
- I didn't want my family or friends to talk to a doctor	1.7% (19)	1.7% (15)	1.6% (4)	
Did the providers or nurses give your family or someone close to you all the information they needed to help you recover?				
- Yes, definitely	32.0% (366)	34.7% (315)	21.3% (55)	<0.001
- Yes, to some extent	35.8% (410)	36.5% (331)	33.7% (87)	
- No	27.4% (314)	23.9% (217)	40.3% (104)	
- No family or friends were involved	1.9% (22)	1.7% (15)	3.5% (9)	
- My family or friends didn't want or need information	2.9% (33)	3.3 (30)	1.2% (3)	
Did a member of staff explain the purpose of the medicines you were to take at home in a way you could understand?				
- Yes, completely	47.2% (539)	48.8% (441)	41.5% (107)	0.004
- Yes, to some extent	30.3% (346)	29.3% (265)	34.1% (88)	
- No	10.8% (123)	9.5% (86)	16.3% (42)	
- I didn't need an explanation	3.2% (37)	3.5% (32)	1.9% (5)	
- I had no medicines	8.4% (96)	8.9% (80)	6.2% (16)	
Did a member of staff tell you about medication side effects to watch for when you went home? (only if question above ≠ I had no medicines)				
- Yes, completely	26.4% (273)	29.1% (238)	16.7% (40)	<0.001
- Yes, to some extent	24.3% (252)	25.2% (206)	19.7% (47)	
- No	43.8% (453)	40.1% (328)	58.2% (139)	
- I didn't need an explanation	5.5% (57)	5.5% (45)	5.4% (13)	
Did someone tell you about danger signals regarding your illness or treatment to watch for after you went home?				
- Yes, completely	37.2% (424)	39.8% (360)	26.4% (68)	<0.001
- Yes, to some extent	31.3% (357)	31.6% (286)	30.2% (78)	
- No	31.6% (360)	28.5% (258)	43.4% (112)	

*P*-values were determined using a Chi-square analysis comparing responses between US and non-US participants.

Based on responses to the Picker Patient Experience questionnaire, the majority of survey participants were satisfied with their healthcare team, feeling that providers and nurses communicated answers to important questions (agreement, providers 86.9% and nurses 86.5%); addressed anxieties and fears about their condition or treatment (78% agreement); were available to discuss concerns for them (70.4% agreement) and their family (72% agreement); and gave them the appropriate information for post-discharge care (67.8% agreement), medications (77.5% agreement), and warnings signs (68.5% agreement). Conversely, one-third of participants disagreed that they received appropriate information about post-discharge care and warning signs. Survey respondents also reported positive experience of care while in the hospital, with most reporting they were treated with dignity and respect (89.1% agreement) and that providers didn't talk in front of them as if they weren't there (68.1%). However, more than two thirds of our survey respondents overall wanted to be more involved in decisions about their care and treatment (68.1%) and despite 80% of them reporting that they were in pain, only 55.7% responded Yes, definitely, 28.4% responded Yes, to some extent, and 15.9% reported that the hospital staff did not do everything they could to help control the pain (the questionnaire does not identify the cause of the pain).

When we explored differences between survey respondents within and outside the US, there were some notable significant differences on some important dimensions of their care experience. Differing from the US participants, participants outside of the US reported less often being able to always get answers that they could understand from a provider (40.6% vs. 27.6%, *p* < 0.01) and from nurses (34.5% vs. 25.4%, *p* < 0.01) and many reported that answers among providers differed often (23.1% vs. 35.0%, *p* < 0.01) and that providers talked in front of them as if they weren't there (28.8% vs. 43.6% reporting agreement, *p* < 0.01). There were also significant differences in the experience of post-discharge preparation, reporting lower provision of appropriate information for post-discharge care (71% vs. 55% Yes, definitely and Yes, to some extent, *p* < 0.01), medication side effects (54.6% vs. 36.4% Yes, definitely and Yes, to some extent, *p* < 0.01), and warnings signs (71.4% vs. 56.6% Yes, definitely and Yes, to some extent, *p* < 0.01).

## Discussion

Virtual communities and social media have increasingly become sources of social support and health information. The PEHSS group demonstrates and supports other research findings that have shown that social media is perceived by mothers as credible sources of parenting and health information (32). Additionally, the specificity of communities such as the PEHSS group allow members to find tailored information with 24-h support, which is not always available from their healthcare providers. Increasingly, researchers are testing social media as a platform for health interventions from prevention of cardiovascular disease to mental health programs to promote overall health and wellbeing (33, 34).

Moderators described experiences of feelings of isolation and challenges with mental health that prompted people to seek the social and emotional support of the PEHSS group as well as the resources and educational materials available. These descriptions are aligned with research that characterizes experiences of PreE as “a condition of uncertainty,” that enhanced ambivalence, confusion, and the need for continuous information (35). In another study, the lack of clear understanding of the signs and symptoms of PreE was widespread, as well as what would differentiate warning signs of PreE from “normal” pregnancy changes (36). This is reflected in approximately one-third of our survey respondents reporting that no one told them about danger signals regarding their illness or treatment to watch for after they went home. This confusion is exacerbated by the perceived lack of information provided by medical professionals to pregnant and postpartum people and their families, highlighted by the results of our survey of PEHSS group members.

Our survey reflected issues with communication between providers and patients and their families and about medical decision making. Notably, two thirds of respondents reported that providers and nurses provided conflicting information about their healthcare and one-third reported not receiving appropriate information for discharge care. These results are similar to findings from a 2006 survey of patients diagnosed with a hypertensive disorder of pregnancy; 68.6% of the respondents were not satisfied with the medical information they received, and respondents were particularly dissatisfied when they suffered from PreE (36). The 2006 study had concluded that patients with disorders such as PreE need to be better informed about their disease and its current and future potential effects. They also found that providers should confirm patient understanding.

Our study results expand these findings regarding patient satisfaction to include patient decision-making. Two thirds of the participants reported wanting to be more involved in their healthcare decisions. For example, proposed medical plans should be presented congruently between all members of the healthcare team to address the issue of conflicting information identified in the survey (37). When the recommended plans change, the reasoning for the change in health management plans needs to be clearly conveyed to patients and their families. By ensuring that all members of the patient's healthcare team have the same, updated information, the patients are able to make better informed decisions.

The consistent lack of understandable and timely medical information, together with unsatisfactory experiences with medical care during pregnancy and delivery, suggest significant opportunities for improvement on the part of the healthcare system overall. A recent randomized control trial found no difference in preeclampsia knowledge before and after education with a graphic card, educational video, or routine prenatal care (37). Even when education tools and resources exist (38), there is a dearth of research identifying evidence-based best practices or implementation strategies to ensure widespread adoption across systems and providers, nurses, and midwives.

### Limitations

Our research had some limitations, including potential response bias from both the moderators in the interviews and the survey participants from the PEHSS group overall. That is, people who join the PEHSS group and who chose to complete the survey may have a had a worse experience than those who did not which could impact the survey results. This may limit the generalizability of our findings, despite the diversity in membership of PEHSS group. An additional limitation is the 1,000+ h/month volunteer effort required to moderate the group, which may not be feasible in the healthcare system. Further, there was limited diversity in survey participant characteristics which could be addressed at least in part in future research with translation to additional languages. Finally, the Picker survey instrument was not specific to hypertensive disorders of pregnancy, which may have limited the sensitivity to the experiences of the participants, and exclusively concerned in-patient experience so does not account for pregnancy or post-partum care.

## Conclusion

While our findings may not be generalizable to other conditions, the research does clearly indicate consistent experiences across geographic groups. Future research is needed to identify best practices in health education and communication around hypertensive disorders of pregnancy. Our research found that the PEHSS group provided emotional and social support and facilitated personal health advocacy for its members, while mental health was identified as a major concern for members. The survey results indicate a global issue in providing support for people with hypertensive disorders of pregnancy during their hospitalization. The needs currently supported through communities on social media highlight opportunities to address critical gaps in care.

## Data Availability

Aggregate data supporting the conclusions of this article will be made available by the authors based on reasonable request.
